# Adenovirus-mediated anti-AEG-1 ScFv expression driven by stathmin promoter inhibits tumor growth in cervical cancer

**DOI:** 10.1186/s12935-020-1159-5

**Published:** 2020-03-12

**Authors:** Min Long, Fang Lin, Xi Wang, Xi Chen, Li Liu, Huizhong Zhang, Ke Dong

**Affiliations:** Department of Medical Laboratory, Tangdu Hospital, Airforce Military Medical University, Xinsi Road, Xi’an, 710038 Shaanxi China

**Keywords:** AEG-1, ScFv, Adenovirus, Stathmin promoter, Cervical cancer

## Abstract

**Background:**

*Astrocyte*-*elevated gene*-*1 (AEG*-*1)* is over-expressed in many cancer cells and has multiple key functions in tumor initiation and progression. Currently, targeted-AEG-1 siRNA is one of the most common techniques to down-regulate AEG-1 expression, but the lack of tumor specificity and available delivery system make it difficult to enter clinical trials.

**Methods:**

In this study, we creatively developed an adenovirus-mediated anti-AEG-1 single-chain antibody fragment (ScFv) expression system driven by a tumor specific promoter, and experimented with it in human cervical carcinoma cells to investigate the effect on tumor’s proliferation and apoptosis.

**Results:**

The results showed that of HeLa and SiHa cells treated with this recombinant anti-AEG-1 ScFv adenovirus not only inhibited cell growth, but induced apoptosis both in vitro and in vivo. Furthermore, we also observed that the expressions of several apoptosis-related genes like Akt 1 and c-Myc decreased, while NF-κB (p65) and cleaved caspase 3 increased on protein levels in vivo.

**Conclusion:**

We concluded that stathmin promoter-driving anti-AEG-1 ScFv adenoviral system may be a breakthrough for its dual-specificity, and serve as an adjuvant tumor specific therapy method in the treatment for human cervical cancers.

## Background

Astrocyte-elevated gene-1 (AEG-1), also called mataderin (MTDH) or LYRIC, was firstly cloned in human fetal astrocytes infected by HIV in 2002 [1]. Subsequently, it becomes increasingly hot in the field of tumor research, for the expression level of AEG-1 is abnormally high in various kinds of cancers [2–4] while extremely low in normal tissues, which suggests that AEG-1 may involve in tumorigenesis and represent a valuable biomarker. Further, many scientists have proposed that AEG-1 might be a novel prognostic marker on the basis of their investigating the correlation between the expression levels of AEG-1 and tumor samples with different clinical stages, whose studies supported that over-expression of AEG-1 was usually associated with lymph nodes/distant metastasis and a poor prognosis [5–7]. Importantly, loss and gain of function studies revealed that AEG-1 involved in multiple signal transduction pathways, and one of the most classic signal pathways was that PI3K-Akt and GSK-3β acted on Ha-ras to activate c-Myc, which subsequently induced AEG-1 to over-express and contributed to AEG-1 exerting its oncogenic properties [8]. Moreover, it has been reported that high-level of AEG-1 is relevant to chemoresistance and down-regulating AEG-1 expression makes tumor cells vulnerable to cancer drugs like cisplatin and pemetrexed [9, 10]. Considering results of recent studies, AEG-1 has emerged as a crucial mediator in tumor malignancy and a key converging point of a complex network of oncogenic signaling pathways. However, shRNA or RNAi, as one of the most common techniques to silence AEG-1, lack in tumor specificity and effective delivery system, which has restricted the further functional exploration for AEG-1.

Specific single-chain variable antibody fragment (ScFv) is one kind of minisize antibody. It is formed by variable heavy chain and light chain domains with a polypeptide linker, which contains the intact antigen-binding site. And owing to its high specificity and affinity for targeted antigen, ScFv could be multifunctional in medicine. For one thing, it is considered to be an available vector when labeled with radionuclides [11], biotoxin or drugs, which will enhance the capability for localizing tumor or the cytotoxic effect on targeted cell. And for another, ScFv has been extensively used as a kind of special drug in treatment of disease. It has been reported that some researchers apply ScFv for infectious disease and tumor [12–14], the results of which offer a promising prospect for clinical application. In recent years, ScFv for a targeted tumor-associated antigen has also been used for constructing CAR-T by combining with the activating machinery of T cell, and the chimeric antigen receptors (CARs) was expressed on T cells. Noticeably, CAR-T with anti-CD19 ScFv has been translated into a clinical product to treat several kinds of leukemia [15].

In our study, we developed a novel adenovirus-mediated anti-AEG-1 ScFv expression system driven by stathmin promoter, the dual-specificity of which is respectively from anti-AEG-1 ScFv and tumor specific promoter. Subsequently, we experimented with the recombinant adenovirus system to block the function of AEG-1 in human cervical carcinoma cells. And we observed proliferation and apoptosis both in vitro and in vivo, meanwhile, we examined the expression of several apoptosis-related gene, AEG-1, c-Myc, NF-κB (p65), Akt_1_ and cleave caspase 3 on protein levels in vivo. Our experimental data will provide evidences for the potential value of adenovirus-mediated anti-AEG-1 ScFv expression system driven by stathmin promoter as an adjuvant tumor-specific therapy method for human cervical cancer.

## Methods

### Preparing anti-AEG-1 ScFv gene

The cDNAs of the variable heavy chain (VH) and light chain (VL) regions of anti-AEG-1 monoclonal IgG transcripts were synthesized by RT-initiated PCR from hybridoma cells secreting the anti-human AEG-1 monoclonal antibody, which the cells and the VH/VL sequences had gained patent protection in China (Patent No.: ZL201210017457.4). Then VL and VH chains were ligated into a single fragment by utilizing the flexible linker (GGGGS). His sequence was inserted into the C-terminal of VH sequence as a tag [16], and the fusion gene was named as AEG-1 ScFv. To evaluate the expression of recombinant anti-AEG-1 ScFv, we then inserted the ScFv gene into pcDNA3.1 vector between *ECORI* and *XHO1* enzyme sites, and the recombinant vector was named as pSFv.

### Construction of stathmin promoter driving AEG-1 ScFv adenovirus

Recombinant stathmin gene promoter driving pDC315 shuttle plasmid named as pDC315-sta was previously obtained by our laboratory [17]. AEG-1 ScFv gene was inserted into pDC315-sta vector between ECORI and SalI enzyme sites to construct stathmin promoter driving AEG-1 ScFv adenovirus, and the recombinant vector was named as pDC315-sta-SFv, and confirmed by both restriction endonuclease digestion analysis and DNA sequencing. The recombinant shuttle plasmids were then co-transfected with the packaging plasmid pBHGloxΔE1, 3Cre into HEK293E cells using Lipofectamine™ 2000 reagent (Invitrogen, Carlsbad, CA, USA) according to the manufacturer’s protocols. pDC315-sta plasmid vector was used as a negative control. After homologous recombination in HEK293E cells, the stathmin promoter driving AEG-1 ScFv adenoviruses were obtained and named as Ad-sta-SFv, meanwhile the negative control adenoviruses was also obtained following the same procedure as above and named as Ad-sta. The recombinant Adenovirus titers in plaque-forming units were then determined by plaque formation assay following infection of HEK293E cells. The multiplicity of infection was defined as the ratio of the total number of plaque-forming units to the total number of cells that were infected. We titrated adenoviruses from duplicate samples in order to confirm the reproducibility of the experiments and the virus titers could reach 1 × 10^11^ pfu/ml.

### Cells infection with recombinant AEG-1 ScFv adenovirus

HeLa and SiHa cells were plated into 6-well plates the day before infection at a seeding density of 5 × 10^4^ cells per well and maintained until the cells reached to 70–80% confluence. The culture medium was discarded and the cells were infected with recombinant adenoviruses of Ad-sta-SFv and Ad-sta negative control at multiplicities of infection (MOIs) ranging from 10 to 300, MOI = 50 group was selected for further functional assay after detection of AEG-1 ScFv expression effect by both PCR and western blot methods. After incubating for 48 h, the cells were washed, trypsinized and collected for future use. These adenovirus infected cells were named as HeLa or SiHa/Ad-sta-sFv and HeLa or SiHa/Ad-sta, respectively.

### RT-PCR and western blot analysis

Total RNA was extracted from the cells, and cDNA was gained by using M-MLV reverse transcriptase (Invitrogen) to amplify AEG-1 ScFv and stathmin gene. For loading normalization, the housekeeping gene β-actin was also amplified from each sample. The PCR primer sequences were as follows: AEG-1 ScFv (833 bp), forward, 5′-*ATGGATGTTTTGATGACCCAAACTCC*-3′; reverse, 5′-*CTAGCCAGGGGCCAGGGGATAAACGGGTGG*-3′. stathmin (323 bp), forward, 5′-AGAACTGGAGAAGCGTGCC; reverse, 5′-CCATTTGTGCCTCTCGGTT. β-actin (160 bp), forward, 5′-*GACTTAGTTGCGTTACACCCTTTC*-3′, reverse, 5′-*TG CTGTCACCTTCACCGTTC*-3′. RT-PCR products were electrophoresed through a 1.5% agarose gel with Ethidium Bromide and signals were quantified by densitometric analysis using the Multilmage™ Light Cabinet (Alpha, USA). For western blot analysis, the recombinant adenoviruses infected cells and control cells were harvested by suspension in lysis buffer at 4 °C for 30 min. The protein concentrations were determined using the Bio-Rad protein assay kit (Bio-Rad, CA, USA). The cell extract (25 µg of total protein) was then analyzed by SDS-PAGE and Western blot. The primary antibodies used in this experiment were antibodies for β-actin, ScFv (anti-Ig Ƙ chain antibody), cleaved caspase 3 (Asp^175^), cleaved PARP (Asp^214^), Bcl-2 and Bax. Densitometry analysis was performed by photoimage analysis using the MultilmageTM Light Cabinet (Alpha, San Leandro, CA, USA). β-actin was also conducted as a loading control and the results were expressed as protein/β-actin absorbance ratio.

### In vitro cell proliferation assay (MTT)

The recombinant adenoviruses infected cells (HeLa/Ad-sta-sFv) and blank control cells (HeLa/Ad-sta) were seeded into seven 96-well culture plates at a seeding density of 2 × 10^4^ per well. Absorption value was detected by MTT (3-(4,5-Dimethyl-thiazol-2-yl)-2,5-Diphenyltetrazolium bromide) at 490 nm after cultivation for 12 h, 24 h, 36 h, 48 h and 72 h, respectively. The proliferation curve of each group was plotted on the basis of absorption values. The inhibitory rates of cell proliferation were calculated according to the following formula: inhibitory rate = (1 − SFv or blank) × 100%. All experiments were performed in triplicate and results were statistically analyzed and expressed as mean ± SD (*n *= 3).

### In vitro colony formation assay

Approximately 3.0 × 10^2^ HeLa cells and pSFv infected HeLa cells were plated in 100 mm culture dishes, respectively. After 14 days, cells were fixed with methanol and stained with 0.1% crystal violet. Visible colonies (> 50 cells) were manually counted.

### Caspase-3 activity assays

Cells were washed with PBS and resuspended in lysis buffer on ice for 15 min. After centrifugation, the supernatants were collected and the caspase-3 activities were measured using the Caspase-3 Activity Assay Kit (Beyotime, Shanghai, China) according to the manufacturer’s instructions. The working principle of this kit is based on the cleavage of the caspase-3 substrate, Ac-IETD-pNA, and the caspase-3 substrate, Ac-DEVD-pNA. The release of p-nitroanilide (pNA) was qualified by determining the absorbance at 405 nm using a microplate reader.

### Flow cytometry analysis

For apoptosis detection, cells were pelleted, suspended in Annexin V-fluorescein isothiocyanate (0.5 mg/ml) and propidium iodide (0.6 mg/ml), and then analyzed with WinMDI software (The Scripps Institute, La Jolla, CA, USA) on FACSCalibur system.

### Nude mouse xenograft studies in vivo

In vivo tumor growth assay was tested in the mouse model by subcutaneous injection of HeLa cells into 8-week-old female athymic nude mice. When ~ 100 mm^3^ tumors were observed, mice were randomly selected for treatment with PBS, Ad-sta and Ad-sta-SFv. Each group consisted of 5 mice. Mice were given recombinant adenoviruses by intra-tumor injection of 1 × 10^9^ pfu (in 100 μl of PBS) of the indicated adenovirus or PBS only once every 3 days for 1 month. The length and width of the subcutaneous tumors as well as the mouse weight were measured every 3 days with calipers. The tumor volume (mm^3^) was calculated with the following formula: *a*^2^*b*/2, where *a* is the short diameter (mm) and *b* is the long diameter (mm). The average tumor size was expressed as mean ± SD (n = 5). After the mice were sacrificed, the tumors were harvested and weighted, and the tumor tissues were homogenized and then analyzed by SDS-PAGE and Western blot. The primary antibodies used in this experiment were antibodies for β-actin, c-Myc, NF-κB (p65), AKt1, p-Akt(Thr^308^), p-Akt(Ser^473^) and cleaved caspase 3 (Abcam, Cambridge, UK). Goat anti-mouse or anti-rabbit IgG coupled to horseradish peroxidase (Santa Cruz) was used as secondary antibodies for detection of protein expression.

### Statistical evaluations

All statistical analyses were performed by using SPSS17.0 (IBM Company, Chicago, USA). Data were expressed as mean ± SD. Comparisons among all groups were performed with the one-way analysis of variance test and Student–Newman–Keuls method. Differences were considered significant at P < 0.05.

## Results

### Recombinant AEG-1 ScFv expressed in cervical cancer cells

**Fig. 1:**
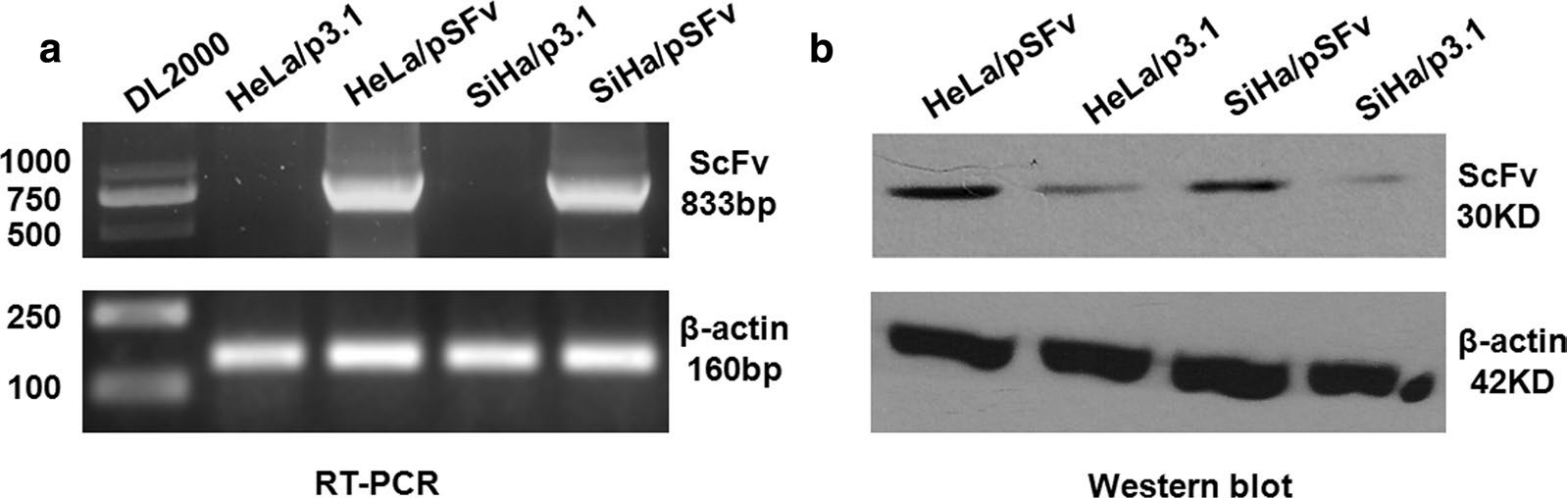
AEG-1 ScFv expression detection. **a** Strong positive bands of AEG-1 ScFv gene in HeLa and SiHa cell lines transfected with pSFv vector were detected by RT-PCR, while negative results were observed in the cells with control vector. **b** AEG-1 ScFv expression detection by western blot, and the results were consistent with above RT-PCR

To confirm that the anti-AEG-1 ScFv fusion gene could express correctly within the cervical tumor cells, we constructed recombinant AEG-1 ScFv expression plasmid pcDNA3.1-SFv and the control vector, and then transfected these two vectors into HeLa and SiHa cells, respectively. The results of RT-PCR and western blot showed that AEG-1 ScFv expressed both on mRNA and protein levels Fig. 1a, b.

### Stathmin gene overexpressed and stathmin gene promoter demonstrated high transcriptional activity in cervical cancer cells

**Fig. 2:**
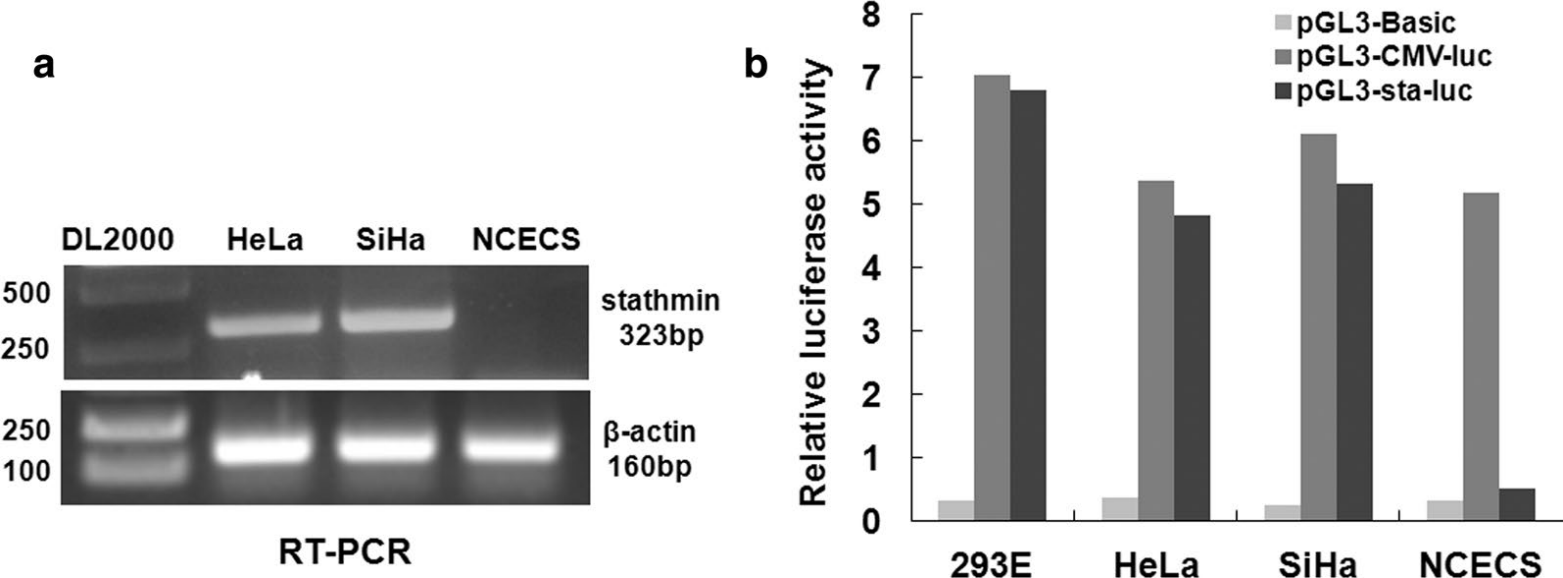
Stathmin mRNA expression and stathmin promoter activity detection. **a** Strong positive bands of stathmin gene in human cervical carcinoma cells HeLa and SiHa were detected by RT-PCR, while negative result was observed in normal human cervical epithelial cells NCECS. **b** Stathmin promoter activity detection by luciferase reporter vectors. Cells were transfected with different luciferase reporter vectors of pGL3-sta-luc (with 451 bp stathmin promoter inserted), pGL3-CMV-luc (positive control) and pGL3-basic (negative control). pGL3-sta-luc showed high activity only in two cervical carcinoma cells compared with pGL3-CMV-luc and pGL3-basic. The histograms represent the average of three independent experiments

To realize the tumor specific and targeted ScFv expression, we firstly, verified the tumor specific activity of stathmin gene promoter and tumor specific expression of stathmin gene both in cervical carcinoma cell lines and normal epithelial cell line by RT-PCR method. As shown in Fig. 2a, stathmin gene was highly expressed in mRNA levels in both of the two cervical cancer cell lines (HeLa and SiHa) but not in normal cell lines (NCECs). Then we tested stathmin promoter activity by transient transfection of pGL3-luc reporter vectors into the three different cells. The activity of the CMV promoter in each cell line was considered as positive control. As shown in Fig. 2b, the stathmin promoter demonstrated high transcriptional activity in HeLa and SiHa cells, but showed nearly background level of transcriptional activity in NCECs cells. These results demonstrated that stathmin promoter driving anti-AEG-1 ScFv expression in Adenovirus was theoretically workable.

### Anti-AEG-1 ScFv expression adenovirus driven by stathmin promoter in cervical cancer cell

**Fig. 3:**
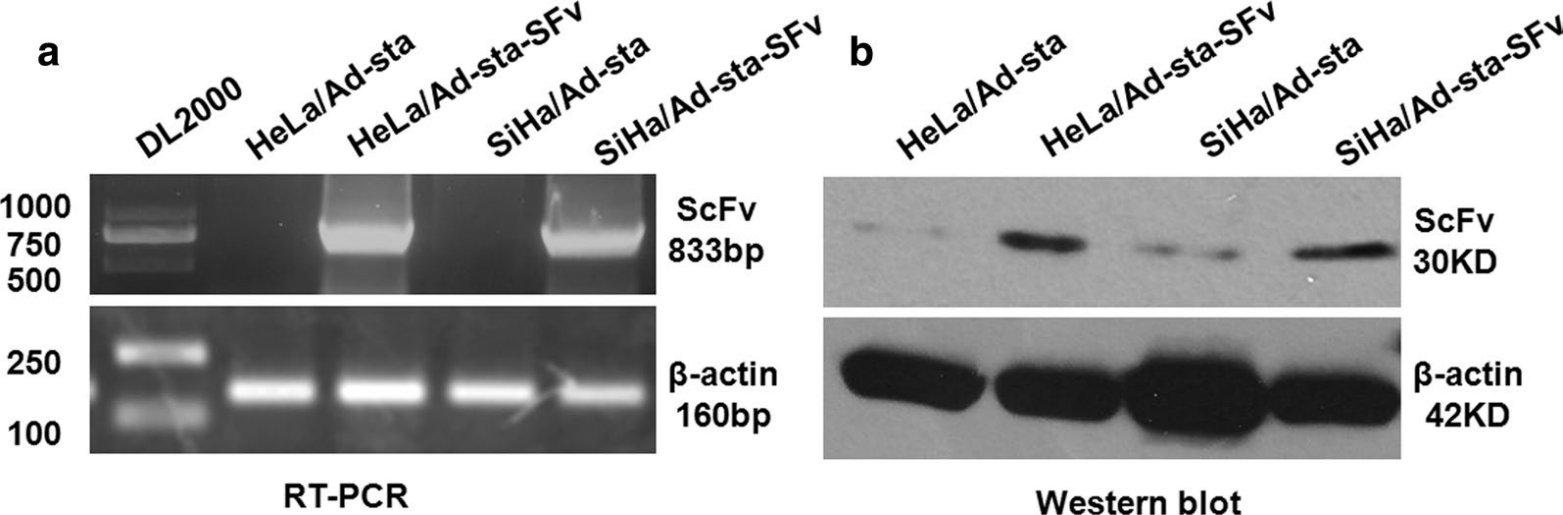
The expressions of anti-AEG-1 ScFv in the cells that infected with recombinant adenovirus. **a** Strong positive bands of anti-AEG-1 ScFv gene were detected in HeLa and SiHa cell lines infected with SFv recombinant adenovirus by RT-PCR, while negative result was observed in cells infected with control viruses. **b** Anti-AEG-1 ScFv expression detection by western blot, and the results were as the same as above RT-PCR

To prepare anti-AEG-1 ScFv expression Adenoviruses, we co-transfected Ad-sta-SFv with pBHGlox△E1, 3Cre packaging plasmid into HEK293 cells, and successfully harvested the recombinant viruses with the titers of 1 × 10^11^ pfu/ml. The expressions of anti-AEG-1 ScFv in HeLa and SiHa cells that infected with recombinant adenovirus could be detected both by RT-PCR and western blot as shown in Fig. 3a, b.

### Anti-AEG-1 ScFv inhibited cell growth and induced cervical cancer cell apoptosis

**Fig. 4:**
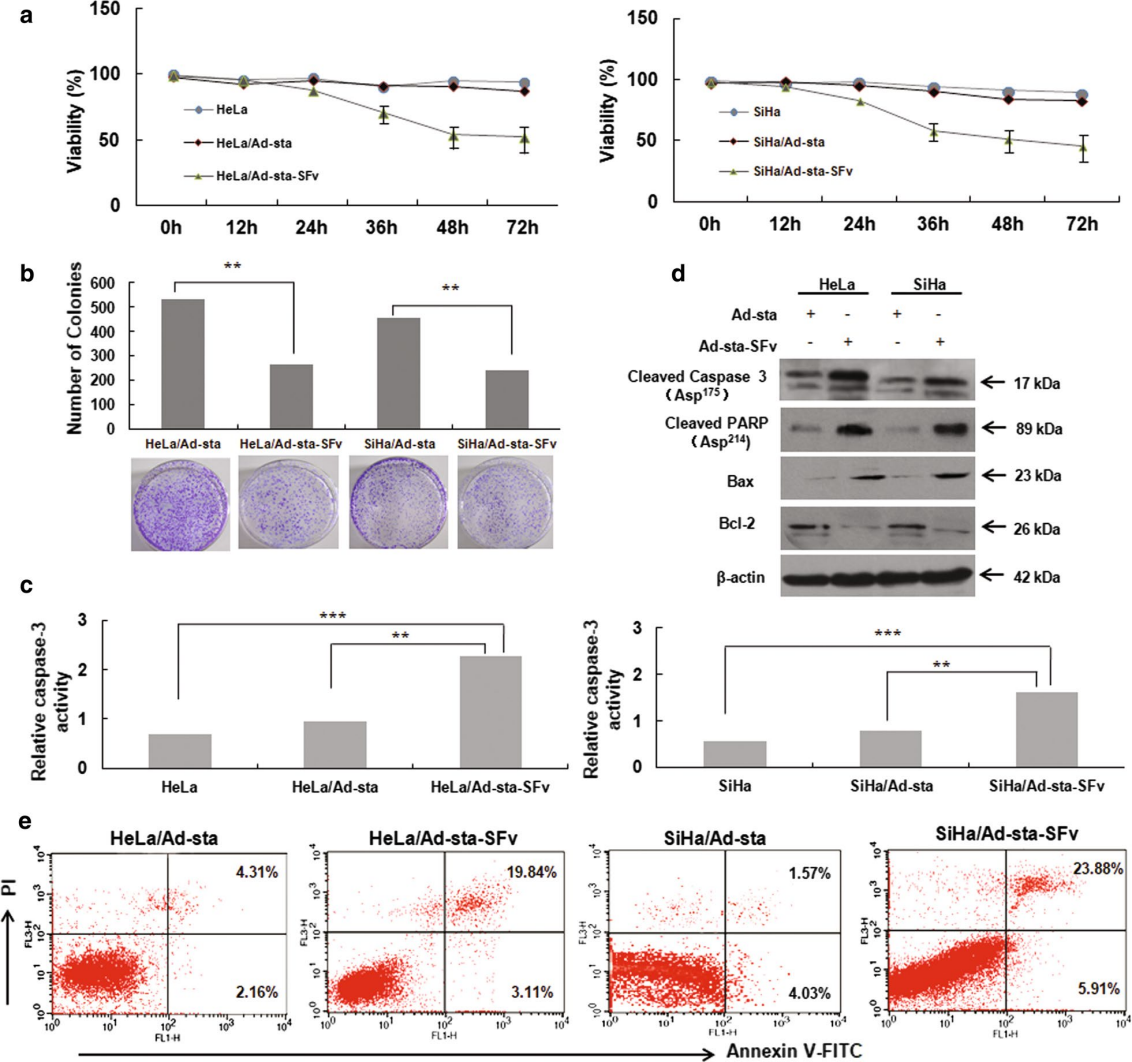
Anti-AEG-1 ScFv inhibited cell growth and induced cervical cancer cell apoptosis. **a** Cell viability detected by MTT assay. Blocking AEG-1 functions by anti-AEG-1 ScFv in HeLa and SiHa cells followed by a subsequent decrease in cell proliferation by 34.99% and 37.46%, respectively, at 72 h compared with the control cells (P < 0.05). **b** Colony formation assay. The number of colonies in the HeLa/Ad-sta-SFV and SiHa/Ad-sta-SFv cell groups (averaged colony number 265 and 239, respectively) was significantly reduced compared with uninfected HeLa or SiHa cells. The experiments were repeated thrice. **P < 0.05. **c** Caspase-3 activities detection. The results showed that the presence of cleaved caspase was markedly detected in Hela/Ad-sta-SFv and SiHa/Ad-sta-SFv compared with control groups, respectively, **P < 0.05, ***P < 0.01. **d** Western blot analysis for the indicated proteins. **e** Apoptosis was determined by by Flow Cytometry assay

To further investigate the effect of Ad-sta-SFv on tumor cells growth in vitro, we performed MTT and colony formation assay with different recombinant adenoviruses treated cells, respectively. The results of in vitro cell proliferation analysis (Fig. 4a) showed that blocking AEG-1 functions by anti-AEG-1 ScFv in HeLa and SiHa cells followed by a subsequent decrease in cell proliferation by 34.99% and 37.46%, respectively, at 72 h compared with the control cells. As expected from the results of MTT assay, the number of colonies in the HeLa/Ad-sta-SFV and SiHa/Ad-sta-SFv cell groups (averaged colony number 265 and 239, respectively) was significantly reduced (P < 0.05) compared with uninfected HeLa or SiHa cells (Fig. 4b). To further investigate whether apoptosis was involved in this process induced by Ad-sta-SFv, we performed the experiment of Annexin V-FITC/PI staining by Flow Cytometry, and also detected the caspase-3 activities by Caspase 3 detection kit. Meanwhile, the expressions of Bcl-2, Bax, cleaved caspase 3 (Asp^175^) and cleaved PARP (Asp^214^), the substrate of the caspase 3, were examined by western blot methods in HeLa/Ad-sta-SFv and SiHa/Ad-sta-SFv cells. The results showed that the presence of cleaved caspase was markedly detected in HeLa/Ad-sta-SFv and SiHa/Ad-sta-SFv cells compared with control cells (Fig. 4c), meanwhile the Western blot results showed that Ad-sta-SFv resulted in a significant decreased expressions of cleaved caspase 3 (Asp^175^) and cleaved PARP (Asp^214^) as well as the Bax levels, while the level of Bcl-2 decreased significantly (Fig. 4d). Flow cytometry analysis showed increased apoptosis in both cell types after transfection by Ad-sta-SFv (19.84% in HeLa cells, 23.88% in SiHa cells, respectively (Fig. 4e). All these results further confirmed that Ad-sta-SFv could efficiently induce cervical carcinoma cells apoptosis via AEG-1 ScFv expression and further inhibition of AEG-1 function by anti-AEG-1 ScFv.

### Ad-sta-SFv inhibited tumor growth in vivo

**Fig. 5:**
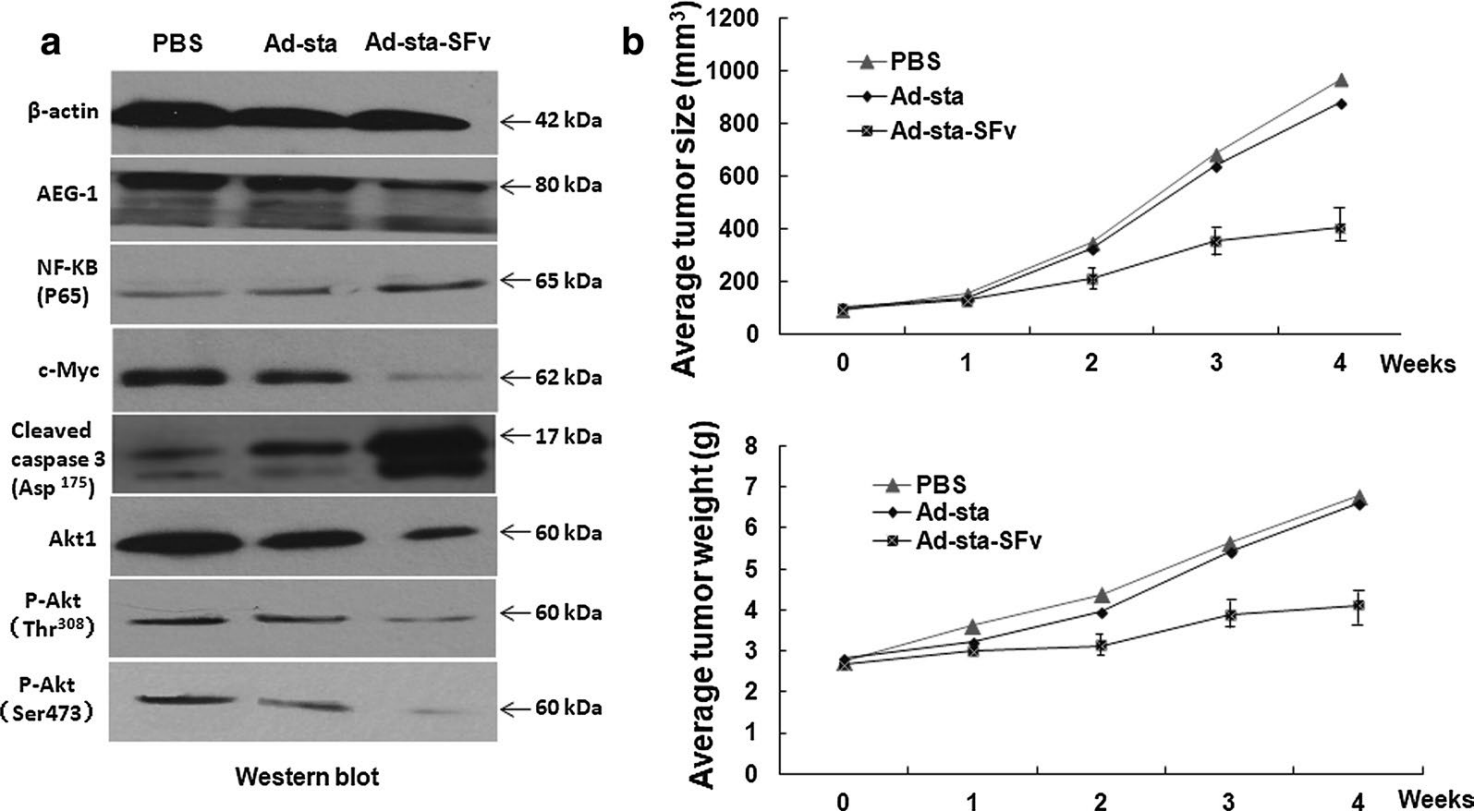
Xenograft tumor growth inhibition results. **a** The expressions of indicated proteins. Apoptosis-related oncogenes like Akt1, p-Akt (ser473) and p-Akt (Thr308) c-Myc were decreased while NF-κB (p65), cleaved caspase 3 increased on protein levels. **b** The tumor growth inhibition experiments results: The inhibition effect was demonstrated by comparing the size/weight of dissected tumors from each group after 4 weeks treatment, respectively, and each bar represents the tumor size/weight of mean ± SD of 10 animals per group. Significant difference from PBS group and Ad-sta group were represented

In order to confirm the therapeutic effect of the recombinant anti-AEG-1 ScFv adenoviruses, we carried out in vivo tumor growth inhibition experiment in nude mice After 4 weeks of inoculation of HeLa cells and treatment with Ad-sta-SFv, we measured tumor size and found that the average size and weight of the xenografts formed from HeLa/Ad-sta-SFv group (averaged size/weight inhibit rate 53.98% and 37.48%, respectively) was obviously smaller than that of the xenografts formed from control groups (Fig. 5b). In the mean while, to further explore the possible mechanism of anti-AEG-1 ScFv on tumor growth inhibition in vivo, we also detected intracellular expression changes of AEG-1, Akt1,c-Myc, NF-ƘB and cleaved Caspase 3 as well as tumor cell apoptosis in Ad-sta-SFv treated tumor tissues. The results showed that the cleaved Caspase 3 (Asp175) and NF-ƘB protein levels were significantly increased while Akt1, p-Akt (ser473) and p-Akt (Thr308) as well as the c-myc expressions were obviously decreased in HeLa xenograft tumortissues treated with Ad-sta-SFv compared with control groups (Fig. 5a), which was accordant with the in vitro results. Taken together, all these results demonstrated that the treatment of AEG-1 ScFv exerted a strong effect on the inhibition of cervical tumor growth in vivo.

## Discussion

As we all known, AEG-1 plays a pivotal role in promoting tumor proliferation and progression, thus many genetic techniques have been applied for downregulating AEG-1’s expression, particularly siRNA. And according to results of current studies, targeted-AEG-1 siRNA is truly effective in inhibiting tumor growth and inducing apoptosis. But it seems difficult that targeted-AEG-1 siRNA could enter clinical trials due to lacking of targeted-tumor specificity and available delivery systems, which urgently calls for a novel way to downregulate expression, or function of AEG-1 and interfere with the interaction between AEG-1 and its ligands.

In our study, we creatively developed an adenovirus-mediated anti-AEG-1 ScFv expression system driven by stathmin promoter. Actually, due to its antigen-targeting and high-affinity, ScFv is supposed to be widely deployed involving various aspects, especially tumor targeting therapy [13, 14, 18]. A recent study has displayed a good result of ScFv in AML that ScFv against EphA2 have specific cytotoxicity effect on AML with EphA2 overexpression [13]. Therefore, we produced a panel of hybridoma cells secreting anti-human AEG-1 monoclonal antibody to construct anti-AEG-1 ScFv, and then we firstly confirmed that recombinant AEG-1 ScFv could express normally in HeLa and SiHa cells by establishing a recombinant anti-AEG-1 ScFv expression plasmid of pcDNA3.1-SFv (Fig. 1a, b).

Considering that AEG-1 mainly located in cytoplasm and nuclear, we presented that adenovirus system may be an ideal vector to escort anti-AEG-1 ScFv into cells. In fact, it has been reported that the combination of ScFv and adenovirus system had been used in tauopathies, which demonstrated that anti-tau ScFv could markedly reduce the level of hyperphorylated, aggregated tau in brain and detergent-soluble tau species in vivo [19]. And this research finding provided our idea with feasibility to bring adenovirus-mediated AEG-1 ScFv into the treatment for cervical cancer.

Furthermore, we improved adenovirus with tumor specific promoter of stathmin gene promoter, which is one of Pol II promoters that displayed high activity in human cancer cells but not in the normal differentiated cells. And as Fig. 2 shown, not only stathmin overexpress in cervical cell lines, but stathmin promoter shows high transcriptional activity compared with normal cell lines (NCECs). In our previous study, the improved adenovirus was used for constructing an adenovirus-mediated Aurora A shRNA driven by stathmin promoter, the loss-of-function results of which showed it could suppress tumor growth and enhance paclitaxel chemotherapy sensitivity in human breast carcinoma cells, more importantly not detrimental to normal cells [17].

Previously, we has proven that downregulation of AEG-1 by shRNA could decrease the levels of p-Akt (Ser^473^) and pAkt (Thr^308^) (the active forms of phosphorylated Akt1) mediated by AURKA inactivation in AML cells, which led to a significant decrease in AML proliferation and increase in apoptosis [20]. Moreover, the similar phenomenon has been observed in cervical cell lines based on our early study that knocking down AEG-1 by shRNA could inhibit SiHa cell proliferation, even invasive ability [21]. To further improve the potential use of AEG-1 target in the treatment of malignant tumor, we packaged an adenovirus-mediated AEG-1 ScFv driven by stathmin promoter and successfully transduced into HeLa and SiHa cells in our present study (Fig. 3). By using these cell models compared with control, we found the cell proliferation of HeLa/Ad-sta-SFv and SiHa/Ad-sta-SFv cells was altered, and obviously decrease, which indicated that Ad-sta-SFv could inhibit cell growth (Fig. 4a). Additionally, Flow cytometry analysis showed increased apoptosis in both cell types after transfection by Ad-sta-SFv (19.84% in HeLa cells, 23.88% in SiHa cells, respectively, as well as the increasing activity of caspase-3 in HeLa/Ad-sta-SFv and SiHa/Ad-sta-SFv cells has been indicated that blocking AEG-1 could induce tumor cell apoptosis. Meanwhile, the significant increased expressions of cleaved PARP (Asp214), the substrate of the caspase 3, further validated the above results (Fig. 4c–e). It is noteworthy that adenovirus-mediated AEG-1 ScFv driven by stathmin promoter is similarly effective, not inferior to targeted-AEG-1 siRNA at least in vitro. For further study, we established three groups of cervical cell carcinoma mouse models which were given recombinant adenoviruses and controls by intra-tumor injection once every 3 days for 1 month, and by comparing the tumor volume and weight among groups, we confirmed in vivo that xenograft tumor cells apoptosis and proliferation inhibition could be induced by using Ad-sta-ScFv.

The malignance of tumor driven by AEG-1 has been reported via several signal pathways. And in our study, we observed in xenograft tumor cells that Ad-sta-SFv could decrease the expression of several apoptosis-related gene, c-Myc, NF-κB (p65), Akt_1_, p-Akt (ser473) and p-Akt (Thr308) and cleaved caspase 3 on protein levels in vivo by blocking the function of AEG-1, which forcefully demonstrated Ad-sta-SFv could accelerate cell apoptosis and cause tumor growth inhibition.

As all known, the ubiquitin proteasome system regulates the turnover of a number of proteins and plays an essential role in maintaining normal cellular function. Dysregulation of ubiquitin-mediated proteolysis results in the development of a variety of human cancers [22]. Our previous studies showed that AEG-1 was also subject to regulation via the ubiquitin proteasome pathway by interaction with the E3 ubiquitin ligase of FBXW7, which plays an important role in mediating the ubiquitination and subsequent proteolytic turnover of protein substrates. The turnover of AEG-1 by FBXW7 led to proliferation arrest and apoptosis in cancer cells [23]. Furthermore, we propose that AEG-1 ScFv could represent a new pathway for the regulation of cell proliferation and apoptosis in cancer cells through negative regulating of AEG-1 levels.

Nowadays, the strategy of ‘one size fit all’ in cancer therapy on the basis of the types and stages of cancer has fallen short in achieving the desired therapeutic index. The role of tumor microenvironment and status of immune system often affect the outcome of therapy. By engaging immune system of the body, primarily macrophages, it is possible to enhance the therapeutic outcome. Induction of bystander killing in cancer cells through drug-exposed cancer cells as well as TAMs is likely to be more effective and conceived as an attractive strategy to uproot tumors [24]. Also, it is of translational importance to devise strategies that “evoke site-specific” by using a drug that is known to preferentially accumulate in tumor cells. Targeted immunotherapy has become a popular research topic in cancer. However, an antibody can itself be utilized as a drug for immunotherapy as well as be employed as a carrier to selectively deliver a large number of therapeutics to cancer cells by recognition of a specific tumor marker. As an ideal vector as well as targeted drug, ScFv has reduced immunogenicity, and can enhance antibody specificity and tissue penetrability and also could be constructed by fusion with other effective molecules, such as chemotherapeutics, compounds, cytokines, nucleic localization signals, biotoxins, and other multifunctional molecules [25, 26]. In our present study, the stathmin promoter-driving AEG-1 ScFv adenoviral system is endowed with dual-specificity, accurately blocking AEG-1 functions and merely targeting tumor cells, so that it is supposed to significantly outperform targeted-AEG-1 siRNA in practicality for translational medicine since such an adenoviral system can precisely aim at an crucial tumor specific antigen with an effective transportation, as well as greatly avoid undesirable side effect, which make AEG-1 ScFv and AEG-1 ScFv fusion complex therapy a promising strategy for the treatment of human malignant tumors.

## Conclusion

In conclusion, stathmin promoter-driving AEG-1 ScFv adenoviral system may have potential value to serve as adjuvant tumor specific therapy method in translation medicine in the treatment for human cervical cancers.

## Data Availability

Not applicable.

## Abbreviations

AEG-1: Astrocyte-elevated gene-1
ScFv: Single-chain antibody fragment
MTDH: Mataderin
VH: Variable heavy chain
VL: Light chain
